# Enhancing life with celiac disease: unveiling effective tools for assessing health-related quality of life

**DOI:** 10.3389/fimmu.2024.1396589

**Published:** 2024-04-29

**Authors:** Ana Luísa Falcomer, Bernardo Romão de Lima, Priscila Farage, Samantha Fabris, Ruth Ritter, António Raposo, Edite Teixeira-Lemos, Cláudia Chaves, Renata Puppin Zandonadi

**Affiliations:** ^1^ Department of Nutrition, Faculty of Health Sciences, University of Brasília, Brasília, Brazil; ^2^ Department of Nutrition, Centro Universitário IESB, Brasília, Brazil; ^3^ Faculty of Nutrition (FANUT), Federal University of Goiás, Goiânia, Brazil; ^4^ CBIOS (Research Center for Biosciences and Health Technologies), Universidade Lusófona de Humanidades e Tecnologias, Lisboa, Portugal; ^5^ CERNAS Research Centre, Polytechnic University of Viseu, Viseu, Portugal; ^6^ ESSV, Centre for Studies in Education and Innovation (CI&DEI), Polytechnic University of Viseu, Viseu, Portugal

**Keywords:** Celiac disease, gluten-free, quality of life, questionnaire, systematic review

## Abstract

Celiac disease (CD) is an autoimmune chronic enteropathy provoked by gluten ingestion in genetically predisposed individuals. Considering it´s only safe treatment is a lifelong gluten-free diet, the burden of living with the disease becomes evident, as well as the need to assess CD health-related quality of life (HRQOL). This review aims to identify and analyze the instruments used to evaluate the HRQOL of adults with CD. This integrative review using a systematic approach was designed to achieve high scientific standards. Accordingly, the search strategy was developed and executed as recommended by the guideline of the Preferred Reporting Items for Systematic Reviews and Meta-Analyses (PRISMA) statement. Detailed individual searches were developed to Pubmed, Science Direct, Scopus, Web of Science, and Google Scholar. After careful analysis of the papers, 43 studies were included, in which seven instruments were identified: Celiac Disease Questionnaire (CDQ) (n=21), Celiac Disease Specific Quality of Life Instrument (CD-QOL) (n=17), Celiac Disease Assessment Questionnaire (CDAQ) (n=4), CeliacQ-7 (n=1), CeliacQ-27 (n=1), Black and Orfila´s self-developed instrument (n=1) and the Coeliac Disease Quality of Life Questionnaire (CDQL) (n=1). The CDQ and CD-QOL were the two most applied instruments. Since the first focuses on the physical and mental symptoms related to the disease and the second focuses on the emotional repercussions of adhering to the GFD treatment for life (dysphoria), the CDQ application is an interesting option for countries that struggle with public policies for CD patients and patients with active CD. The CD-QOL could be used for countries with strict regulations for CD and gluten-free products and populations in remission. When comparing results among different populations, it is preferable to utilize culturally validated instruments, which have been applied across multiple countries, providing greater comparability between study findings.

## Introduction

Celiac disease (CD) is an autoimmune chronic enteropathy by the ingestion of gluten in genetically predisposed individuals. It affects approximately 1% of the world´s population (1). As CD is linked to small bowel mucosa damage, its classic form main feature includes gastrointestinal malabsorption syndrome. Its clinical picture usually includes chronic diarrhea, abdominal distention and pain, weight loss, and failure to grow in infants (2, 3).

However, patients also usually face a wide range of extraintestinal symptoms and disorders that might include chronic fatigue, depression, anxiety, osteoporosis, compromised fertility, and libido, especially in women (4, 5). Although CD ordinarily combines a vast number of symptoms, some individuals are asymptomatic, even in cases in which mucosal damage is present (6). Those patients have a heightened risk of complications since they do not recognize the clinical aggravation of CD and tend to be more resistant to the treatment (7, 8).

Until now, the only safe and effective treatment for CD is the adoption of a gluten-free diet (GFD), characterized by the exclusion of cereal grains (wheat, rye, barley, and, in some cases, oats) and all their derivatives from the diet (9). When following a strict GFD, most patients experience remission of the disease’s physical manifestations and normalization of small bowel mucosa (10, 11). Nonetheless, compliance with the GFD is challenging since it requires changes in lifelong dietary habits, which are accompanied by the lack of information and guidance for the preparation of healthy gluten-free meals among the general population, the high cost of gluten-free products, the risk of gluten cross-contamination, and even social exclusion (12). The combination of the physical, emotional, and social burdens and worries related to the GFD experienced by celiac patients is directly related to how they perceive their quality of life (4, 9).

Quality of life (QOL) is a multidimensional concept that includes subjective evaluations of both positive and negative aspects of life regarding individuals’ goals, expectations, standards, and concerns (13). In the past years, worries about QOL in CD have increased and many questionnaires have been developed, adapted to different cultures, and validated to explore patients’ perception of well-being (9, 14, 15).

However, instruments used to measure the QOL of celiac individuals must be carefully elaborated to comprise the specificities of CD, from its clinical manifestations to the overall difficulties faced regarding compliance with the GFD (16, 17). Thus, it is important to highlight that questionnaires whose domains do not address these particularities may present limitations (18, 19). Therefore, CD population-specific validated questionnaires are the most reliable ones since they include the patients’ struggles and CD specificities (20, 21).

To our knowledge, no studies compare the existing instruments that measure celiac individuals’ QOL, nor the main domains used to evaluate it. In this sense, this review aimed to identify and analyze the instruments used to evaluate the health-related quality of life of adults with celiac disease. The findings of this study may guide researchers in studies related to QOL and assist the development of public policies for celiac individuals, reducing the impact on health assistance and the costs of treatment of CD and its consequences.

## Methods

This integrative review using a systematic approach was designed to achieve high scientific standards. Accordingly, the search strategy was developed and executed as recommended by the guideline of the Preferred Reporting Items for Systematic Reviews and Meta-Analyses (PRISMA) statement (22).

### Eligibility criteria

This review included quantitative studies that developed; translated and culturally adapted; or validated questionnaires to measure the QOL of adults with CD. Studies that evaluated QOL in the population mentioned using instruments designed for people with CD were also included.

All studies that analyzed the QOL of celiac patients using instruments designed for the general population were excluded. Additionally, qualitative studies, studies in which the population evaluated was under eighteen years old, reviews, letters, conference summaries, case reports, short communications, and books were excluded.

### Information sources and search strategy

Detailed individual search strategies were developed for the following databases: Pubmed, Science Direct, Scopus, and Web of Science. Partial gray literature research was conducted using Google Scholar. The final search in all databases was performed on September 7th, 2023. Additionally, a manual examination of the reference lists of the full-text studies included was performed to ensure that possible relevant studies that could have been lost during the electronic search of databases were identified.

The literature search was conducted in English using the following terms, their mesh terms, and synonyms: “quality of life” AND (“celiac disease” OR “coeliac disease”) AND (“questionnaire” OR “instrument”) AND “adults”. The appropriate combinations of truncation and words were selected and adapted to the search specificities of each database (**Supplementary Table S1** - **Supplementary File**). No limitations of language or date of publication were applied; therefore, all studies published until the final search were included.

All references were managed by Endnote Web. After removing duplicate hits, the references were transferred to Rayyan, where the authors performed the selection of titles and abstracts.

### Study selection and data collection processes

**Figure 1:**
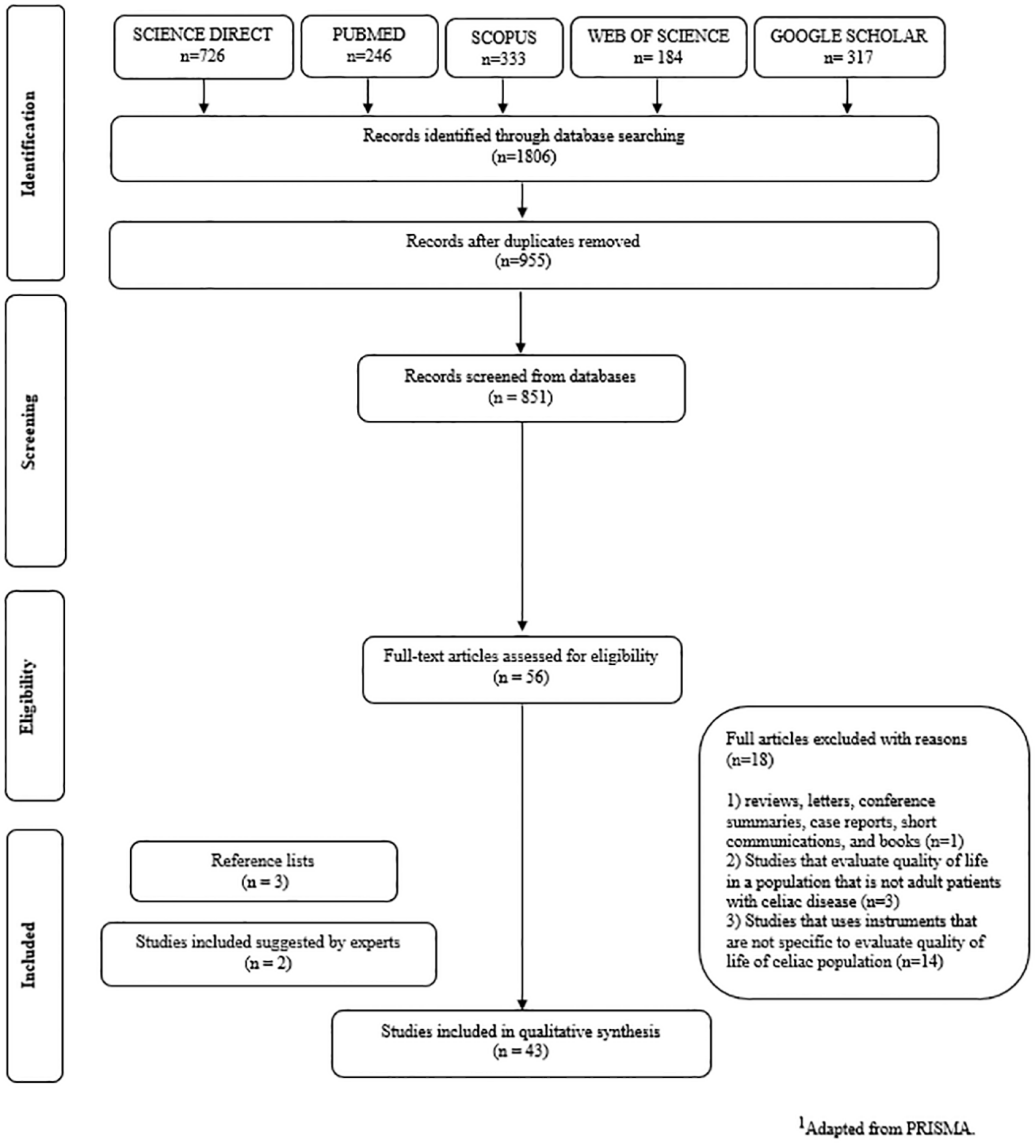
Flow Diagram of Literature Search and Selection Criteria.^1^

Calibration exercises were conducted before starting the review to ensure consistency among reviewers. The selection was conducted in two phases. In phase 1, two reviewers (SF, RR) independently reviewed the titles and abstracts of all references identified from databases. Articles that did not meet the eligibility criteria were discarded. In phase 2, the same reviewers (SF, RR) applied the eligibility criteria to the full texts of the selected articles. In cases of disagreement, the two reviewers discussed until a consensus was obtained. A third reviewer (ALF) made the final decision when there was no consensus. These data were synthesized by the three reviewers (SR, RR, ALF) using a standardized table. The final selection was always based on the complete text of the publication. The list of references from the selected studies was critically evaluated by the BRL examiner. Additional studies were added by the experts (PF and RPZ). **Figure 1** demonstrates the search and study selection processes through a flow diagram.

### Data extraction

The following characteristics were collected from the selected articles and synthesized using a standardized table containing authors and year of publication, the country where the research was conducted, the aim of the study, methods, participants and sample size, instrument(s) used to measure the quality of life, and main findings. The complete table with collected results is available in **Table 1**.

**Table 1:** Summary of descriptive characteristics and instruments included in the studies.

Author	Year	Country	Title	Aim	Study design	Instrument(s) to measure of CD quality of life–
Häuser et al. (17)	2006	Germany	Development and Validation of the Celiac Disease Questionnaire (CDQ), a Disease-specific Health-related Quality of Life Measure for Adult Patients with Celiac Disease.	To develop and validate a disease-specific questionnaire to measure HRQOL in adult patients with Celiac Disease.	Development and validation of questionnaire	CDQ
Häuser et al. (15)	2007	Germany	Predictors of reduced health-related quality of life in adults with coeliac disease.	To test predictors of reduced health-related quality of life, described in the literature, by a multivariate approach	Cross-sectional study	CDQ
Dorn et al. (19)	2010	United States of America	The development and validation of a new coeliac disease quality of life survey (CD-QOL)	To develop and psychometrically validate a new coeliac disease-specific instrument, the CD-QOL	Development and validation of questionnaire	CD-QOL
Zampieron et al. (23)	2011	Italy	Quality of life in adult celiac disease in a mountain area of Northeast Italy	The aim of this study was to evaluate the health-related quality of life in patients diagnosed as having celiac disease and to study the factors involved in its impairment of quality of life	Cross-sectional study	CDQ
Black et al. (24)	2011	United Kingdom	Impact of coeliac disease on dietary habits and quality of life	The study aimed to investigate the effect of CD and a GFD on dietary habits and quality of life of a cohort of adult biopsy diagnosed coeliac patients who reside in England.	Cohort	Self-developed questionnaire
Zingone et al. (25)	2013	Italy	The Italian translation of the celiac disease-specifc quality of life scale in celiac patients on gluten free diet.	To assess the validity and reliability of the Italian translation of the Celiac Disease-specific Quality of Life Scale	Translation and cultural validation of a valid questionnaire	Italian version of CD-QoL
Marchese et al. (26)	2013	Italy	Quality of life in coeliac patients: Italian validation of a coeliac questionnaire.	To translate, cultural adapt and perform validation of the CDQ for use in Italy.	Translation and cultural validation of a valid questionnaire	Italian version of CDQ
Casellas et al. (27)	2013	Spain	Transcultural adaptation and validation of the Celiac Disease Quality of Life (CD-QOL) survey, a specific questionnaire to measure quality of life in patients with celiac disease	To translate and validate in Spanish the specific celiac disease questionnaire CD-QOL.	Translation and cultural validation of a valid questionnaire	Spanish version of CD-QOL
Lobão et al. (28)	2013	Portugal	Development of the Portuguese Version of the Celiac Disease Questionnaire	To develop the Portuguese version of the Celiac Disease Questionnaire - CDQ (developed by Dr. Winfried Häuser team in 2007	Translation and cultural validation of a valid questionnaire	Portuguese version of CDQ
Pouchot et al. (29)	2014	France	Validation of a French Version of the Quality of Life ‘‘Celiac Disease Questionnaire’’	The objectives of this study were to provide a cross-cultural adaptation of the specific quality of life ‘‘Celiac Disease Questionnaire’’ (CDQ) and to analyze its psychometric properties	Translation and cultural validation of a valid questionnaire	French Version of CDQ (F-CDQ)
Casellas et al. (20)	2015	Spain	Benefit on health-related quality of life of adherence to gluten-free diet in adult patients with celiac disease	To examine the effect of adherence to the GFD on health perception of celiac patients measured using a specific questionnaire.	Cross-sectional study	Spanish version of CD-QOL
Castilhos et al. (5)	2015	Brazil	Quality of live evaluation in celiac patients from southern Brazil	This study aimed to evaluate the quality of life of patients with celiac disease on a capital in Southern Brazil	Cross-sectional study	CD-QOL
Aksan et al. (30)	2015	Turkey	Validation of the Turkish version of the Celiac Disease Questionnaire (CDQ)	The aim of the study was to translate, adapt and validate the Celiac Disease Questionnaire (CDQ), which was developed in Germany, for use in Turkey	Translation and cultural validation of a valid questionnaire	Turkish Version of CDQ
Lee et al. (31)	2016	United States of America	Coeliac disease: the association between quality of life and social support network participation	To exam the association between participation in different types of social support networks and quality of life in adults with CD	Cross-sectional study	CD-QOL
Rodríguez-Almagro et al. (32)	2016	Spain	Health-related quality of life and determinant factors in celiac disease. A population-based analysis of adult patients in Spain	To determine the health-related quality of life in a representative sample of Spanish adults with celiac disease along with its determinant factors.	Cross-sectional study	Spanish version of CD-QOL
Mahadev et al. (33)	2016	United States of America	Quality of Life in Screen-detected Celiac Disease Patients in the United States	To determine if differences exist between screen-detected and symptom-detected CD patients with regard to measures of QOL and dietary adherence.	Cross-sectional study	CD-QOL
Lee and Clarke (34)	2017	United States of America	Effect of clinical and laboratory parameters on quality of life in celiac patients using celiac disease-specific quality of life scores	To investigate the association between HR-QOL and clinical, laboratory findings using the previously validated CD-QOL (celiac disease-specific quality of life) instrument in patients with celiac disease.	Cross-sectional study	CD-QOL
Dowd and Jung (35)	2017	Canada	Self-compassion directly and indirectly predicts dietary adherence and quality of life among adults with celiac disease	To examine self-compassion in relation to celiac specific quality of life (CQoL) and adherence to a GFD among adults with celiac disease.	Cross-sectional study	CD-QOL
Skjerning et al. (11)	2017	Denmark and Ireland	A comprehensive questionnaire for the assessment of health-related quality of life in coeliac disease (CDQL)	To develop the Coeliac Disease Quality of Life questionnaire (CDQL): a comprehensive CD-specific HRQoL measure that can be completed by children, adolescents, and adults or by proxy.	Development and validation of questionnaire	CDQL
Real-Delor R. E. and Centurion-Medina I. C.	2017	Paraguay	Quality of life in adults from paraguay with celiac disease	The objectives of this research were to determine the quality of health-related life in adolescents and adults with celiac disease and to investigate conditions that they affect it.	Cross-sectional study	Spanish version of CD-QOL
Zysk et al. (9)	2018	Poland	Social and Emotional Fears and Worries Influencing the Quality of Life of Female Celiac Disease Patients Following a Gluten-Free Diet	The aim of the study was to analyze the social and emotional fears and worries influencing the QoL of female CD patients following a gluten-free (GF) diet, as well as to indicate the sociodemographic interfering factors.	Cross-sectional study	CDQ
Crocker et al. (36)	2018	United Kingdom	Quality of life in coeliac disease: qualitative interviews to develop candidate items for the Coeliac Disease Assessment Questionnaire	To gain indepth understanding of the impact of CD on HRQoL from the perspective of adults with the condition.	Questionnaire development	CDAQ
Crocker et al. (37)	2018	United Kingdom	Quality of life in coeliac disease: item reduction, scale development and psychometric evaluation of the Coeliac Disease Assessment Questionnaire (CDAQ)	To develop a questionnaire in accordance with best practice guidelines, capturing all aspects of quality of life important to adults with coeliac disease	Development and validation of questionnaire	CDAQ
Pratesi et al. (21)	2018	Brazil	Quality of Life of Celiac Patients in Brazil: Questionnaire Translation, Cultural Adaptation and Validation	The study aimed to translate, culturally adapt and validate a celiac disease quality of life questionnaire and apply it to a representative number of Brazilian CD patients	Translation and cultural validation of a valid questionnaire	Brazilian version of CDQ
Barzegar et al. (38)	2018	Iran	Transcultural Adaptation and Validation of Persian Version of Celiac Disease Questionnaire (CDQ); A Specific Questionnaire to Measure Quality of Life of Iranian Patients	The aim of this study was to validate a Persian version of Celiac Disease Questionnaire (CDQ) for Celiac disease (CD) among Iranian patients.	Translation and cultural validation of a valid questionnaire	Persian Version of CDQ
Burger et al. (39)	2019	Netherlands	How to best measure quality of life in coeliac disease? A validation and comparison of disease-specific and generic quality of life measures	To search for a brief, reliable, and valid tool to accurately assess the relevant quality of life domains in patients with coeliac disease. In addition, to investigate whether a disease-specific HRQoL questionnaire would add relevant information to a generic HRQoL questionnaire to better identify patients experiencing problems.	Translation and cultural validation of a valid questionnaire; Development and validation of questionnaire.	Dutch version of CD-QOL - CD-QOL-NL; self-developed instrument - CeliacQ-27; and its shorted version - CeliacQ7.
Crocker, H; Jenkinson, C; Peters, M;	2020	United Kingdom	Healthcare experiences and quality of life of adults with coeliac disease: a cross-sectional study	To investigate patients´s experiences of healthcare services in coeliac disease, from before diagnosis to the time of the survey, as well as explore the relationship between experiences of healthcare and quality of life.	Cross-sectional study	CDAQ
Harnett and Myers (40)	2020	Australia	Quality of life in people with on going symptoms of coeliac disease despite adherence to a strict gluten-free diet	To report on the quality of life in this specifc group of patients, with CD who have persistent symptoms despite adherence to a gluten free diet.	Cross-sectional study	CDQ
Fueyo-Diaz et al. (41)	2020	Spain	The effect of self-efficacy expectations in the adherence to a gluten free diet in celiac disease	To investigate the role of general and specific self-efficacy and their relationship with other psychosocial variables that can affect adherence to a GFD in patients with CD.	Cross-sectional study	Spanish version of CD-QOL
Casellas et al. (42)	2020	Spain	National survey on the experiences of people with celiac disease in Spain. The CELIAC-SPAIN project.	To know the opinion of patients and relatives regarding different aspects related to celiac, the unmet needs in the diagnosis and management of the disease, as well as the difficulties that patients have in following the diet.	Cross-sectional study	Spanish version of CD-QOL
Selleski (43)	2020	Argentina	Evaluation of Quality of Life of Adult Patients with Celiac Disease in Argentina: From Questionnaire Validation to Assessment	To translate, culturally adapt, validate, and apply the CDQ to a representative sample of the celiac population in Argentina.	Translation and cultural validation of a valid questionnaire	Argentinian version of CDQ
Fueyo-Díaz et al. (41)	2020	Spain	Influence of Compliance to Diet and Self-Efficacy Expectation on Quality of Life in Patients with Celiac Disease in Spain.	To study the adherence to the GFD and HRQoL in patients with CD in Spain.	Cross-sectional study	Spanish version of CD-QOL
Muhammad et al. (44)	2021	United Kingdom	Telephone clinic improves gluten-free dietary adherence in adults with coeliac disease: sustained at 6 months	To evaluate the effect of a telephone clinic on GFD knowledge and GF dietary adherence in adults with CD.	Case-control	CDAQ
Falcomer et al. (45)	2021	Brazil	Health-Related Quality of Life and Experiences of Brazilian Celiac Individuals over the Course of the Sars-Cov-2 Pandemic.	To evaluate Brazilian celiac patients’ QoL during the pandemic caused by the outbreak, rapid spread, and subsequent restrictive measures caused by COVID-19, in addition to the dietary restrictions and other burdens caused by CD.	Cross-sectional	Brazilian version CDQ
Schiepatti et al. (46)	2021	Italy	Long-Term Adherence to a Gluten-Free Diet and Quality of Life of Celiac Patients After Transition to an Adult Referral Center.	The aim of the study is threefold (1): to provide an overview on the clinical features, long-term GFD adherence, QOL, and continuity of follow-up of patients diagnosed with CD during childhood/adolescence and then followed-up at an adult tertiary referral center for CD over a twenty-year period (2); to evaluate whether timing of transition impacts long-term GFD adherence, QOL, and continuity of follow-up; and (3) to identify predictors of long-term GFD adherence in adulthood.	Prospective cohort	Italian version of CDQ
Szőcs et al. (47)	2021	Hungary	Shame mediates the relationship between stigma and quality of life among patients with coeliac disease.	The main aim of the study was the adaptation of the SSCI-8 and the necessary psychometric testing among celiac women. In addition, the study also aimed to investigate the relationship between stigmatization and different well-being variables among celiac women.	Cross-sectional study	CDQ
Dimidi et al. (8)	2021	United Kingdom	Predictors of adherence to a gluten-free diet in celiac disease: Do knowledge, attitudes, experiences, symptoms, and quality of life play a role?	To identify the relationship between adherence to a GFD and demographic characteristics, knowledge, attitudes, and beliefs regarding CD and a GFD, experiences of following a GFD, symptoms, and QoL.	Cross-sectional study	CDQ
Parada et al. (48)	2021	Chile	Adherence to a gluten-free diet and quality of life in Chilean celiac patients	To evaluate adherence to GFD and its relationship with quality of life in Chilean celiac patients.	Cross-sectional study	Spanish version of CD-QOL
Nikniaz et al. (49)	2021	Iran	The Persian Translation and validation of the celiac disease quality of life questionnaire (CDQOL)	To translate CDQOL into Persian and evaluate the psychometric properties of the Persian version.	Translation and cultural validation of a valid questionnaire	Persian version of CD-QOL
Moreno et al. (50)	2022	Spain	Quality of Life in Teenagers and Adults with Coeliac Disease: from Newly Spanish Coeliac Disease Questionnaire Validation to Assessment in a Population-based Study	To translate, culturally adapt, validade, and apply the Spanish version and estimate the HRQoL, using the EQ-5D in a representative sample of the Spanish teenagers and adults with CD.	Translation and cultural validation of a valid questionnaire	Spanish version of CDQ
Enaud et al. (51)	2022	France	Compliance with Gluten Free Diet Is Associated with Better Quality of Life in Celiac Disease	To determine the disease and clinical factors associated with better QOL in a large cohort of French CD patients.	Cross-sectional study	French Version of CDQ (F-CDQ)
Guennouni et al. (52)	2022	Morocco	Quality of life of Moroccan patients with celiac disease: Arabic translation, cross-cultural adaptation, and validation of the celiac disease questionnaire.	to translate, cross-culturally adapt, and validate the items of the CDQ and eventually evaluate the QoL among adults with CD in Morocco	Translation and cultural validation of a valid questionnaire	Morrocan version of CDQ
Chaves et al. (53)	2023	Portugal	Quality of Life Perception among Portuguese Celiac Patients: A Cross-Sectional Study Quality of Life Perception among Portuguese Celiac Patients: A Cross-Sectional Study Using the Celiac Disease Questionnaire (CDQ)	To assess Portuguese celiac patients’ quality of life (QoL) perception.	Cross-sectional study	Portuguese version of CDQ

## Results

After a systematic literature search and subsequent peer analysis, 43 studies, published between 2006 and 2023, were included in this review. **Table 1** presents the studies’ general characteristics.

**Figure 2:**
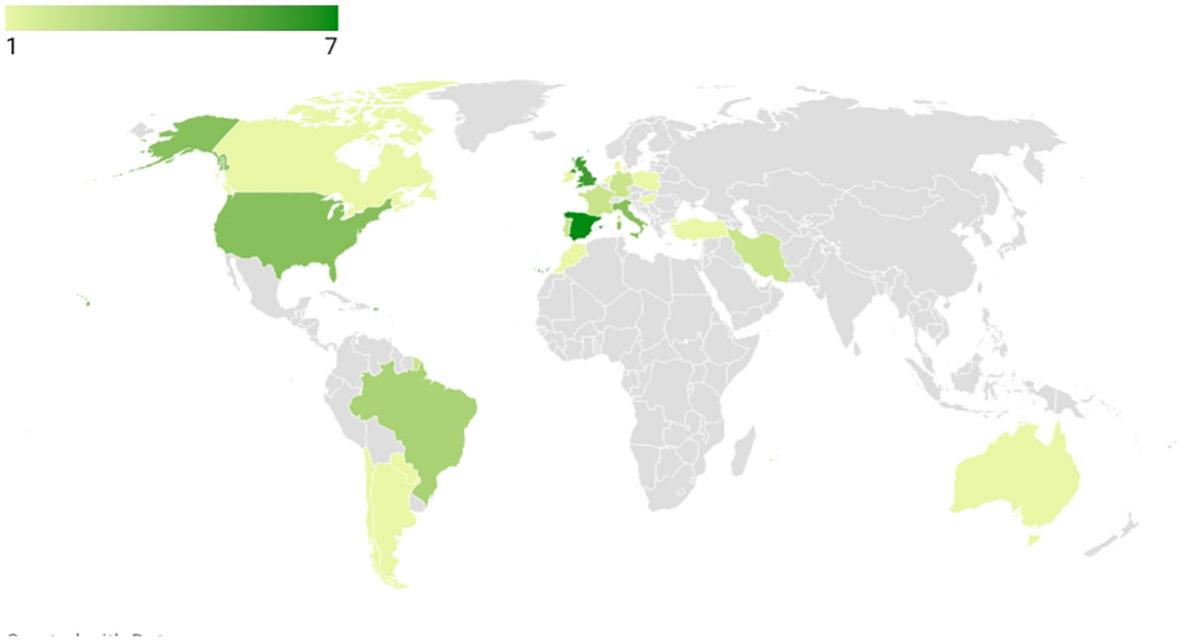
Chroropleth map generated from the number of studies according to their origin. Higher frequencies are represented with darker tones of green.

The 43 included studies were conducted in a total of 21 countries. Spain had the highest number of studies with 16.28% (n=7), followed by the United Kingdom (UK) (13.95%, n=6), the United States of America (USA), and Italy (9.3% each, n=4). In South America, Brazil accounted for 6.98% (n=3) of the studies, while Argentina, Chile, and Paraguay each contributed one study (2.33%). Iran, Portugal, France, and Germany each had two studies (4.65%, n=2 per country). Additionally, Canada, Hungary, Netherlands, Poland, Morocco, Australia, and Turkey contributed one study each (2.33% each). A collaboration between Denmark and Ireland resulted in a joint research contribution (2.33%, n=1). A choropleth map regarding the distribution of the frequencies of included studies by different countries is available in **Figure 2**.

**Figure 3:**
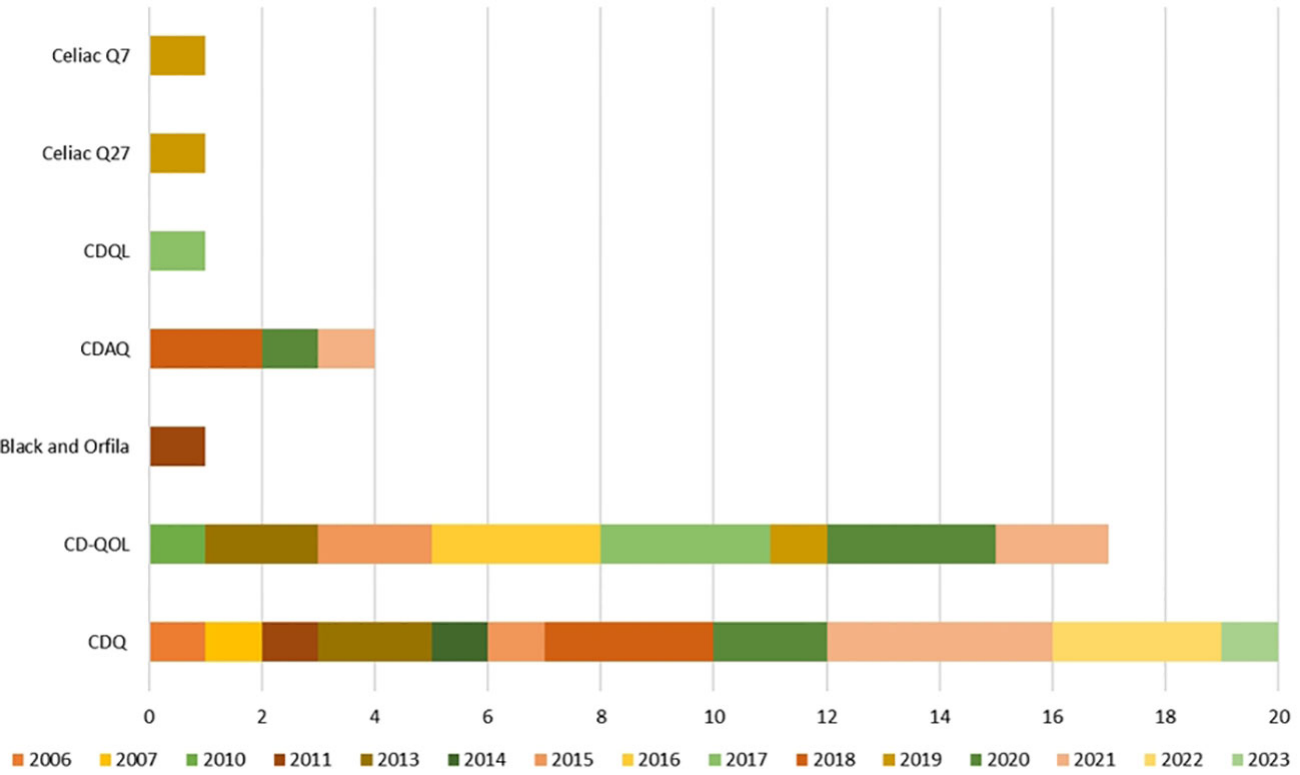
Use of specific questionnaires to measure celiac patients’ quality of life throughout time.

This article comprises information on studies published in a 17-year period, with the majority (53.33%, n=24) published in the last six years (2018-present). As evidenced in **Figure 3**, the first instrument developed to measure celiac QOL, the CDQ, was developed in 2006 (17) and set an important precedent for researchers in the field, since then, it became evident that the assessment of celiac QOL should be performed using tools designed to the celiac population specificities.

Among the studies that applied validated methods and questionnaires for analyzing QOL of CD patients, following instruments were identified: (i) Celiac Disease Questionnaire (CDQ) (n=21) (ii) Celiac Disease Specific Quality of Life Questionnaire (CD-QoL) (n=17); (iii) Celiac Q27 (n=1); (iv) Celiac Q7(n=1); (v) Coeliac Disease Assessment Questionnaire (CDAQ) (n=4); and (vi) Celiac Disease Quality of Life Questionnaire (CDQL) (n=1). It is imperative to emphasize the importance of cultural adaptation when using validated instruments like the ones mentioned in this review in different sociocultural backgrounds since the experiences and challenges faced by individuals with celiac disease can vary across different countries and cultural backgrounds. Studies carried out without cultural adaptation may fail to accurately capture the unique factors influencing the quality of life for celiac patients in the countries´ specific contexts.

**Figure 4:**
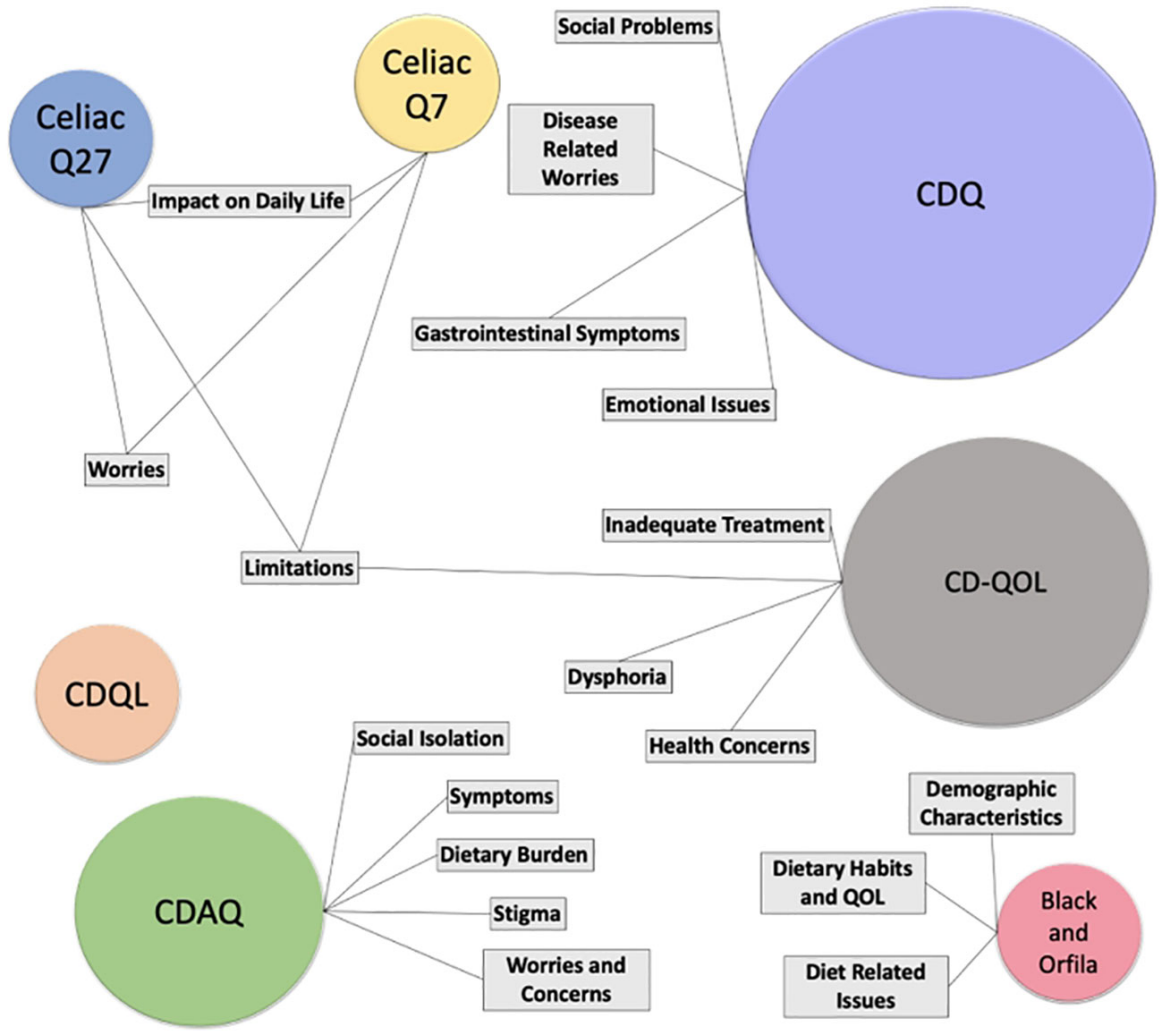
Graphical representation of the present domains in the included instruments and their respective intersections. Most utilized instruments are represented with proportional bigger circles.

Concerning the domains in the included instruments, a total of fifteen domains were observed when all instruments were analyzed together Clique ou toque aqui para inserir o texto (14, 17, 21, 27, 32, 34, 52). **Figure 4** graphically represents the domains of the seven instruments included, it also represents intersections between domains with similar nomenclatures/subjects in different questionnaires.

It is important to note that similar denominations of the domains present in different questionnaires can evaluate different constructs depending on the questionnaire. Also, domains with different nomenclatures evaluate similar constructs.

## Discussion

As displayed in **Figure 2**, the countries that have executed more research on the topic are Spain, followed by the UK, Italy, and the USA. It can be inferred that developed countries tend to have more preoccupation regarding health-related quality of life (HRQOL), more access to CD diagnoses and deal with less misdirection from CD diagnosis due to infectious diseases, which represents a struggle to control acute gastrointestinal cases, and the fact that there used to be a misconceived association of CD with populations exclusively of Caucasian origin (54, 55). This may explain the range of countries where studies regarding the quality of life of celiac patients were conducted.

In addition, it is essential to emphasize the importance of investigating celiacs´ health dimensions in nations that have not yet done so, even though CD has been reported in them, such as India and Russia, both of which rank among the world’s ten most populous countries, along with several others (54, 56). The recent increase in studies reflects a growing awareness about how celiac disease impacts patients’ quality of life. This heightened focus may stem from either an escalating prevalence of CD over time or an increased recognition of its importance as a global public health issue in the past two decades (54, 55).

## CD specific instruments to assess QOL

### Celiac disease questionnaire

The CDQ was designed in Germany in 2007 through the prism of patients, experts in the CD field and scientific literature to evaluate celiac individuals’ HRQOL, being a pioneer in the subject (17, 18). It is a quantitative 10-minute self-administered questionnaire composed of four subscales: emotional issues, social problems, disease-related worries, and gastrointestinal symptoms. Each subscale has seven sub-items each, resulting in a total of 28 questions (18, 30).

The CDQ domains consider the patient´s feelings and perceptions concerning the challenges the disease and the GFD impose in their lives (29).The subcategories comprehend the dimensions of HRQOL, which are physical, emotional and social, and were related to domains of other disease-specific instruments like the Chronic Liver Disease Questionnaire (57) and the Inflammatory Bowel Disease Questionnaire (58).

The participants’ answers are evaluated using a 7-point Likert scale that varies in crescent punctuation, from always, most of the time, often, now and then, rarely, almost never, and never (18). The CDQ final evaluation score is a result of the addition of each question´s points, and therefore ranges from a total of 0 to 196 points; the score varies from 0 to 49 in all domains (18, 30). Lower scores indicate reduced HRQOL, hence higher values indicate high HRQOL (18). Although the CDQ score does not have a cut-off point, its development included a group of people who do not present CD-related disease as a comparison (18).

Throughout various studies conducted in different countries, the CDQ was used for measuring HRQOL in its original version, developed and validated in Germany (17), and has also been translated, culturally adapted, and validated to a diverse range of populations. The adapted versions were applied to populations in Italy (25, 26, 46), Portugal (28, 53), France (29, 51), Turkey, Brazil (21, 45), Poland (9), Iran (38), Argentina (43), Morocco (52), and Spain (50). Furthermore, the original CDQ was employed in studies conducted in Germany (17), Italy (23), Poland (9), Australia (40), Hungary (47), UK (8). Overall, the CDQ has been applied across 21 different studies (48.4%) spanning 15 countries, corresponding to 71,42% of the nations that investigated the thematic and are contemplated in this review.

In the original paper, the total score of QOL indicated that in Germany, people with CD presented lower QOL (143.1) than people without CD-associated disease (157.6), suggesting that celiac does negatively impact patients’ well-being (18). As the CDQ was developed for the German population, performing cultural adaptation as well as translation and validation of the tool to other countries is strongly recommended to minimize bias in QOL assessment and, consequently, data interpretation. In addition, since the original version of CQD is designed for on-paper applications, adapting to a web-based version is interesting for online applications.

The CD populations that presented the highest and lowest CDQ scores were Italian (159 score) (26) and Portuguese (103 score) (53). As the instrument has been applied only once in Portugal, it is not possible to compare the QOL scores over time. However, as the data was collected during COVID-19 pandemic, it could have negatively interfered with the score, especially over the social domain punctuation (53). However, as discussed by the Portuguese study, isolation can have a positive effect on GFD adherence, and it can be analyzed in Brazilian scores (53).

Brazil´s first assessment of celiac QOL was in 2018 and obtained a 119.79 (21) and the second CDQ application took place during COVID-19 and found a 125.26 score (45). Authors associate the improvement in celiac well-being in the pandemic period to the reduced social interactions involving gluten-containing food, which have negative repercussions in social and emotional domains, as well as the increase of home meal preparation that is a protective factor to GFD adherence, impacting positively in the emotional, social, worries and symptoms scales 383838.

Regarding the psychometric quality and quantitative parameters, the Cronbach’s alpha for the CDQ domains ranged from 0.80 to 0.91, as instruments are viable when Cronbach’s alpha is higher than 0.7 (18). All adaptations of the questionnaire presented over 0.7 values of Cronbach’s alpha. Therefore, the CDQ is a valid instrument to measure QOL of celiac patients that contemplates HRQOL dimensions.

### Celiac disease specific quality of life instrument

The CD-QOL is a quantitative, self-administered questionnaire of 20 items distributed across four subscales: limitations, dysphoria, health concerns, and inadequate treatment (19). Researchers sought to capture in the instrument how patients perceive changes in their daily lives after diagnosis by consulting a celiac support group for input during the focus group stage (19). The answers to each item are allocated into a 5-point Likert Scale response, ranging from “not at all” to “a great deal”.

Each subscale is associated with factors that can negatively impact the QOL of individuals with CD. The first factor is called limitations and refers to the feeling of facing difficulties in belonging to or being part of routine events, such as eating with coworkers, not being able to eat conventional foods on important occasions (e.g., birthday cake) or struggling to take long trips due to CD and GFD (19). The second factor is dysphoria and is associated with a feeling of emotional dissatisfaction or discomfort due to the CD, the items in this category question if the participant feels depressed, frightened, or overwhelmed about having CD; it also inquires If the person believes not to have enough knowledge about CD (19). The third subscale, health concerns, is based on items of concern of increased risk of stigma due to the disease (19). The last category is inadequate treatment and it´s items inquire if patients feel like the GFD is sufficient treatment for CD (19).

It is noticeable that the CD-QOL approach focuses more on the individuals’ perceptions of external elements and challenges related to adhering to a gluten-free lifestyle rather than on gastrointestinal or extra gastrointestinal symptoms affecting the quality of life, from an urge to use the bathroom to sexual activities. The main difference between the CDQ and the CD-QOL instruments is that the first focuses on repercussions of the CD in physiological repercussions, psychological symptoms, and impact in daily activities (e.g., work, leisure, etc.); whereas the second targets attitudes and perceptions of the celiac population in routine events such as socializing. The CD-QOL does not include any item to measure the physical impact of CD since the individuals in its population, which included American celiac support group members, did not emphasize symptoms as a struggle, which could be a characteristic of the USA population or public policies. That highlights the need to perform a cultural adaptation and validation of the questionnaire when assessing the QOL of people with CD. The questionnaire was applied in 17 papers (39,53%) of 9 countries and applied in 8 nations (38.09% of all countries included in this review).

The original version of CD-QOL was utilized in the USA (19, 31, 33, 34), Canada (35), Brazil (5). Cultural adaptation and validation were conducted in Italy (25), Spain (20, 27, 32, 41, 42, 59), and Iran (49); the CD-QOL was also adapted to the Netherlands (39), but it was not applied to the population, it was used to develop new questionnaires. The Spanish version of the instrument (27) was applied to the Spanish-speaking countries of Paraguay (60) and Chile (48); however, it was not adapted to South American specificities. It´s possible to suggest that all four subscales of the CD-QOL are susceptible to changes due to regional influences in the exposome and public policies such as regulations for specialized health service support to people with CD, therefore the cultural adaptation is recommended even for countries with same mother language (55).

### CeliacQ-27 and CeliacQ-7

The CeliacQ-27 and CeliacQ-7 aim to evaluate CD HRQOL. It was developed and validated in Dutch, considering the cross-cultural adaptation of the CD-QOL to the Netherlands (19, 39). These questionnaires offer a unique approach by comparing different phases of CD, including active/clinical remission periods as well as instances where individuals may deviate from their gluten-free diet.

The CeliacQ-27 consists of 27 questions categorized into three domains: limitations (11 items), worries (10 items), and impact on daily life (6 items) (39). The limitations domain is related to patients’ perceptions of daily life restrictions because of the CD or GFD. The worries domain questions about mental and general challenges associated with CD (39). The third domain, impact on daily life, comprises questions about the social influences of the CD and GFD (39). Higher scores in de CeliacQ27 equal better QOL. The internal consistency of the questionnaire domains ranged from Chronbach´s α of 0.87 and 0.92, demonstrating good to excellent reliability (39).

Its shorter version contained only seven questions (CeliacQ-7) and was created by excluding all items with loads <0.70 Chronbach´s α in the Dutch version of the CD-QOL (39). The final version obtained a 0.88 Chronbach´s α and a high correlation with the CeliacQ-27 (39).

Since the CeliacQ-27 and the CeliacQ-7 are both derived from the Danish version of the CD-QOL, the two do not include questions to assess the repercussions of the physical symptoms associated with CD in patients’ well-being (39). However, introducing a condensed questionnaire could increase participation rates in surveys and be an interesting tool for ambulatory assistance and follow-ups, especially for patients who have been following a GFD.

### Coeliac disease assessment questionnaire

The Coeliac Disease Assessment Questionnaire (CDAQ) was developed in two stages in 2018 (36, 37). The premise for developing this instrument was that the questionnaires available at the time were constructed without considering patient-reported outcome measures (PROM), which consider patients’ point of view, not necessarily measured by biological markers or associated with clinical outcomes (61).

In the first phase of development, qualitative interviews were conducted and analyzed through a data framework, which revealed six common themes reported by the participants: symptoms, gluten-free diet, emotional health, impact on activities, relationships, and financial issues (36). In the first phase, 64 items were present in the instrument (36). The subsequent phase was centered on the item reduction of the first version of the instrument; in this sense, items were refined through item appraisal, expert review, cognitive interviews and translatability assessment (37).

The resultant instrument comprises 32 items in five domains: stigma, dietary burden, symptoms, social isolation and worries, and concerns (37). Concerning its psychometric quality and quantitative parameters, this instrument presented Cronbach’s alpha between 0.82 and 0.88 for all domains. These values demonstrate the viability of the instrument, since an instrument of this type is considered viable when its Cronbach’s alpha is higher than 0.7 (62). Another highlight regarding CDAQ’s quantitative parameters is related to its strong intraclass correlation (0.86) with SF-36’s domains, the Short Form Health Survey developed by the World Health Organization (WHO) (37).

Regarding its application, the questionnaire is structured on a five-point Likert scale (never, rarely, sometimes, often and always), which is later converted into a scale of 0-100, with 100 being the highest quality of life (QOL) (37). Two studies (4.65%) included in this review used this instrument, one being the pilot study developed by Crocker et al. (2018) (36) in the United Kingdom and another carried out through a telephone survey in the same region in 2020 (44).

In a study of 276 people (166; 61.9% women and 110; 38.1% men), the results revealed an average quality of life score of 53.6 on the instrument´s proposed 0-100 scale (37). Significant differences (p <0.05) were found between the two groups, with male participants showing a higher overall quality of life (60.91) compared to women (49.18). However, potential reasons for this difference were not explored by the authors (37).

The other study that used the CDAQ as an instrument to assess the QOL with CD evaluated the effectiveness of telephone monitoring in improving the QOL of this population, evaluating, in addition to the quality of life, adherence to a GFD (44). The results demonstrated that although the intervention was effective in improving adherence to the gluten-free diet, quality of life parameters did not differ significantly between the treated and control groups, with emphasis on assessments in the “dietary burden” domain, which assesses the difficulty of diet be followed (44).

Based on its quantitative parameters, the CDAQ is an appropriate instrument for assessing the QOL in patients with CD. However, the low application of this questionnaire in studies outside the UK stands out, in addition to the fact that, to date, studies regarding the translation of this instrument into other languages ​​have not been carried out. A possible hypothesis is that the instrument is relatively new (2018) compared to other instruments already developed and applied in different countries, such as the CDQ, which was developed in 2006 (15, 37).

### Coeliac disease quality of life questionnaire

The CDQL consists of a 44-item questionnaire developed in 2017 for CD patients of all ages and applied in one of the studies contemplated in this review (n=1, 2,32%) (11). It was designed in Ireland and Denmark and occurred in three phases: focus groups to collect celiacs’ insights and important aspects of QOL that should be in the final questionnaire; CD patients responded to the pilot version of the CDQL; refinement of the final version of the CDQL and application to Danish participants (11).

The final version of the instrument was web-based and estimated to take 5-10 minutes to complete (11). It included twelve items about background information, covering demographic characteristics and diagnosis data; two generic QOL questions; and 30 CD specific questions attributed to ten scales, the first two being general and followed by eight CD specific categories (11). The CDQL evaluates specific CD scales including: worries-about-symptoms (1 general item with thirteen alternatives); symptoms (1 general item with thirteen alternatives); contacting health care (3 items); having coeliac disease and following a gluten-free diet (7 items); communicating about coeliac disease and gluten-free diet (4 items); others´ handling my coeliac disease (3 items); confronting gluten-containing food (4 items); knowing about coeliac disease and gluten-free food (3 items); gluten-free food supply (3 items); evaluating having coeliac disease in overall (3 items) (11).

For scoring procedures, items are evaluated through a 5-point Likert scale, alternating among ´very unwell´, ´unwell´, ´neutral´, ´well´ or ´very well´. All response choices were followed by a smiley communicating the analogous emotion (11). The final score was calculated using the average score of each scale, higher results on the Likert scale indicate better QOL (11).

Though the CDQL was developed for children, adolescents and adults, the focus group step included only a few adults, which poses a limiting factor despite the items being formulated in a non-age-restricted way (11). On the other hand, this instrument presents the opportunity to evaluate patient reported HRQOL across different ages using a unique questionnaire (11). As far as we know, the CDQL has not been reapplied or translated/adapted culturally to any other country. Further studies are necessary to corroborate the pilot study results and their applicability to all age segments.

### Self-developed questionnaire (Black and Orfila)

One of the studies included in this review (2.32%) aimed to analyze the quality of life of participants in an observational cohort conducted in 2011 with 146 CD patients who were members of the Coeliac UK Charity (24). However, in addition to the fact that this study did not use an instrument created and validated by other authors, it also included a food frequency questionnaire, seeking to relate the quality of life with the participants’ dietary habits (24).

In this sense, the authors developed a questionnaire composed of 32 questions, of which 10 are related to demographic variables, 10 to dietary habits (including availability of gluten-free foods and accidental ingestion of gluten due to cross-contamination) and 12 questions related to quality of life (24). It is important to highlight that although the questionnaire developed by the authors is not validated, such questions were derived from previously validated questionnaires, such as the Canadian Celiac Health Survey and the EPIC-norfolk food frequency questionnaire (63, 64).

It´s structured on a Likert scale, with five points: all of the time, most of the time, some of the time and never (24). Furthermore, the results were interpreted based only on the frequencies of answers on each point, with no corresponding scale to assess the quality of life (24).

As a main result, the cohort study demonstrated that CD impacts participants’ daily habits; however, most participants reported good physical health (24). Also, 97% of the participants reported good dietary compliance, with results supported by the food frequency questionnaire (24). Regarding the critical situations that affect the quality of life of these people, anxiety and depression related to social isolation resulting from dietary restriction and pain resulting from both intentional and unintentional gluten ingestion have been reported (24).

As the main limitation of this instrument, it is important to highlight that given the fact that it has not been validated or culturally adapted, the results from this study cannot be extrapolated to other populations with celiac disease. Also, no statistical assessment of the psychometric constructs of the items and sections was performed.

## Domains of the included instruments

As presented in **Figure 4**, CD symptoms are explicitly described only in the domains of the CDQ (17) and CDAQ (37). However, while the CDQ domain related to symptoms only presents questions regarding bowel movements, diarrhea, gas, bloating and abdominal cramps, CDAQ includes symptoms related to mental health, such as tiredness, exhaustion, limitation of daily activities and general pain (36, 37).

Yet, issues regarding the same symptoms are also assessed in the instrument developed by Black and Orfila (24); however, under both domains of Dietary Habits and QOL and Diet-related issues. In the CDQ (17) instrument, such constructs are assessed under the “emotional issues” domain and in Celiac Q27 and Celiac Q7 in the Impact of Daily Life domain (39).

The “worries” domain is present in the CDQ (17), Celiac Q7 and Q27, CD-QOL (19) and CDAQ (36, 37) instruments. However, different scales relate to which spheres of life such worries refer. While in Celiac Q7 and 27 (39) such worries include conditions that relate to problems such as food availability outside the household, social interactions, and unpredictable bowel movements, in other questionnaires such as the CDAQ (36, 37), CDQ (17), and CD-QOL (19) such conditions are better stratified into other specific domains such as “Dietary Burden”, and “Social Problems”. In the CDAQ instrument, the concern regarding the availability of safe gluten-free food is measured in questions from the “dietary burden” domain (36, 37).

The domain entitled “Limitations” is found in the Celiac Q27 575757, Celiac Q7 (39) and CD-QOL (19) instruments. In both CeliacQ27 and Celiac Q7, questions assigned under this domain regard quotidian challenges present in patients’ lives, such as persistent symptoms, changes in the composition of foods previously labeled as “gluten-free” and situations regarding social acceptance while coexisting with celiac disease (39). In the context of CD-QOL (19), similar questions are present in the limitations´ domain, thus showing similarities between those three instruments.

However, questions regarding similar situations before addressed in the “Limitations” domain in CD-QOL (19), Celiac Q27 and Celiac Q7 (39) are also present in other instruments, for example, in “Dietary Related Issues” domain in the instrument created by Black and Orfila (24), “Disease related worries” (CDQ (17)) and “Social isolation” (CDAQ (36, 37),.

It is important to highlight that evaluating domains is challenging, given the semantic obstacles related to the proposed construct to be evaluated by different instruments (65). Furthermore, given the context that most instruments undergo translation and cultural validation, possible changes in the meaning of the constructs may occur (66, 67). Such differences make it difficult to compare the effectiveness of different instruments which, despite being individually statistically validated, may not be possible to be applied together given the differences between the literal meanings of the domains present (65–67).

The assessment of QOL in individuals with CD plays a crucial role in gaining insight into the well-being and impact of the disease. To effectively measure QoL, researchers have developed and validated various questionnaires that consider the unique experiences and management strategies associated with CD. Notably, two questionnaires stood out, the CDQ and the CD-QOL. Since the first focuses on the physical and mental symptoms related to the disease and the second focuses on the emotional repercussions of adhering to the GFD treatment for life (dysphoria), the CDQ application is an interesting option for countries that struggle with public policies for CD patients and patients with active CD; whereas the CD-QOL could be used for countries that have GF and CD regulations and populations in remission. When comparing results among different populations, it is preferable to utilize culturally validated instruments, which have been applied across multiple countries, providing greater comparability between study findings.

## Author contributions

AF: Conceptualization, Formal Analysis, Investigation, Methodology, Visualization, Writing – original draft, Writing – review & editing. BdL: Formal Analysis, Investigation, Visualization, Writing – original draft, Writing – review & editing. PF: Formal Analysis, Investigation, Methodology, Visualization, Writing – original draft, Writing – review & editing. SF: Investigation, Visualization, Writing – review & editing. RR: Investigation, Visualization, Writing – review & editing. AR: Funding acquisition, Resources, Supervision, Writing – review & editing. ET-L: Funding acquisition, Writing – review & editing. CC: Funding acquisition, Writing – review & editing. RZ: Conceptualization, Formal Analysis, Funding acquisition, Investigation, Methodology, Project administration, Resources, Supervision, Visualization, Writing – original draft, Writing – review & editing.
