# Supplementary material for: Enhancing life with celiac disease: unveiling effective tools for assessing health-related quality of life
Source: Front Immunol. 2024 Apr 29;15:1396589. doi: 10.3389/fimmu.2024.1396589 (PMC11089154; doi:10.3389/fimmu.2024.1396589)
Supplement: Supplementary file 1 [file Table_1.docx]

**Table S1** – Database and terms used for searching the references about specific instruments for measuring quality of life of individuals with celiac disease.

| Database | Search (Sep 07^th^, 2023) |
| --- | --- |
| Pubmed | (("quality of life" OR "welfare" OR "well-being" OR "QoL") AND ("questionnaire" OR "questionnaires" OR "survey" OR "surveys" OR "instrument" OR "instruments") AND ("celiac disease" OR "coeliac disease" OR "Gluten Enteropathy" OR "Gluten Enteropathies" OR "Gluten-Sensitive Enteropathy" OR "celiac" OR "coeliac" OR "celiacs" OR "coeliac") AND ("adult" OR "adults")) |
| Web of science | (TS=("quality of life" OR "welfare" OR "well-being" OR "QoL")) AND (TS=("questionnaire" OR "questionnaires" OR "survey" OR "surveys" OR "instrument" OR "instruments")) AND (TS=("celiac disease" OR "coeliac disease" OR "Gluten Enteropathy" OR "Gluten Enteropathies" OR "Gluten-Sensitive Enteropathy" OR "celiac" OR "coeliac" OR "celiacs" OR "coeliac")) AND (TS=("adult" OR "adults")) |
| Science Direct | (("quality of life" OR "well-being" OR "QoL") AND ("questionnaire" OR "survey") AND ("celiac disease" OR "coeliac disease" OR "celiac") AND ("adult")) |
| Scopus | TITLE-ABS-KEY (( "quality of life"  OR  "welfare"  OR  "well-being"  OR  "CD-QoL"  OR  "QoL") AND ("celiac disease" OR "coeliac disease" OR "Gluten Enteropathy" OR "Gluten Enteropathies" OR "Gluten-Sensitive Enteropathy" OR "celiac" OR "coeliac" OR "celiacs" OR "coeliacs") AND ("questionnaire" OR "questionnaires" OR "survey" OR "surveys" OR "instrument" OR "instruments") AND ("adult" OR "adults")) AND (LIMIT-TO (DOCTYPE, "ar") OR  LIMIT-TO ( DOCTYPE ,  "sh")) |
| Google Scholar | "celiac disease" "quality life" "instrument" |
| Google Scholar | "celiac disease" "quality life" "questionnaire" |
| Google Scholar | "celiac disease" "quality life" "adults" |
